# Downloading appetite? Investigating the role of parasocial relationship with favorite social media food influencer in followers’ disordered eating behaviors

**DOI:** 10.1007/s40519-024-01658-4

**Published:** 2024-04-22

**Authors:** Reza Shabahang, Sohee Kim, Xiuhan Chen, Mara S. Aruguete, Ágnes Zsila

**Affiliations:** 1 Department of Psychology, College of Education, Psychology and Social Work, Flinders University, Bedford Park, SA Australia; 2 Department of Counseling and Instructional Sciences, University of South Alabama, Alabama, USA; 3 University of Missouri, Missouri, MO USA; 4 Department of Social and Behavioral Sciences, Lincoln University, Missouri, MO USA; 5 Institute of Psychology, Pázmány Péter Catholic University, Budapest, Hungary; 6 Institute of Psychology, ELTE Eötvös Loránd Universtiy, Budapest, Hungary

**Keywords:** Eating disorder, Food addiction, Influencer, Parasocial relationship, Social media

## Abstract

**Purpose:**

Although a number of investigations have been carried out on the marketing outcomes of parasocial relationships (PSR) with food influencers on social media, little attention has been paid to the potential contribution of these one-sided emotional bonds to followers’ eating attitudes and habits. Drawing on the *Parasocial Theory*, the role of parasocial attachment with food influencers was investigated in predicting eating disorders, food addiction, and grazing. To increase the accuracy of PSR measurement, a brief self-report scale was developed to gauge social media users’ feelings of mutual awareness, attention, and adjustment with their favorite food influencer at a distance through social media.

**Methods:**

Participants were a convenience sample of 405 Iranian social media users (231women; *M*_*age*_ = 28.16, *SD*_*age*_ = 9.40), who followed a favorite food influencer on social media.

**Results:**

The 8-item *Parasocial Relationship with Favorite Food Influencer Scale* (*PSRFFIS*) revealed a unidimensional structure with excellent content and construct validity and internal consistency. Regarding gender differences, men showed stronger parasocial attachment to their favorite food influencers. Adjusting age, gender, and subjective social status as control variables, PSR with favorite food influencers partially contributed to the explanation of eating disorder symptom severity, food addiction, and grazing.

**Conclusion:**

These findings show that PSR with favorite food influencers appears to be associated with followers’ craving for food, which, in turn, may contribute to maladaptive eating habits. This highlights media-related factors, such as PSR with food influencers, as potential drivers of dysfunctional eating habits in the digital age, particularly in countries like Iran where disordered eating is prevalent.

**Level of evidence:**

Level V—based on cross-sectional data (correlational study; scale development)

## Introduction

Social media influencers as opinion leaders and role models are on the rise, and such figures are acquiring celebrity capital in a saturated media landscape. These media characters can shape followers’ attitudes and behaviors [1]. In some cases, influencers turn into a source of eating inspiration. Several studies have found that influencer’s promotion of food affects users' eating attitudes and preferences [2].

Drawing on the *Parasocial Theory* and extant studies on influencer marketing and influencer–audience attachment, a parasocial relationship (PSR) is recognized as an appreciable constituent of an influencer’s persuasion. *Parasocial Theory* posits that audiences experience imagined social relationships as symbolic and one-sided social-emotional bonds with media persons in their mediated encounters [3]. PSRs apply to connections between social media users and influencers. Studies have offered evidence on the crucial role of PSR on followers' interests, attitudes, and intentions [4]. Therefore, food influencers can possibly shape their followers’ eating attitudes and behaviors.

Changes in media use habits continue to raise questions, with special focus on uncertainties surrounding the impact of modern interactive media on eating behaviors. Most studies have so far focused on the marketing facets of parasocial relationships with food influencers (e.g., purchase intention) rather than the psychological factors associated with this attachment. Scant attention is paid to the contribution of the intensity of PSR with food influencers to users’ maladaptive eating patterns. Furthermore, a large proportion of populations in the Middle East are classified as high risk for disordered eating [5]. In specific, reports have unveiled unexpectedly high prevalence of eating disorders (EDs) in Iran [6]. Meanwhile, the research on media-related risk factors associated with EDs in the Middle East, and specifically within the Iranian population, is scarce. In addition, there is currently no reliable measure of PSR specifically with food influencers in the literature. The aim of this study was to (a) construct and validate a brief scale to quantify PSR with favorite food influencers, (b) explore the demographic correlates of PSR with favorite food influencers, and (c) investigate the relationship of PSR with favorite food influencers with eating disorders, food addiction, grazing, and body-mass-index (BMI).

## Methods

### Participants

A convenience sample of 405 Iranian adult social media users (231 women and 174 men; *M*_*age*_ = 28.16, *SD*_*age*_ = 9.40) were recruited through advertisements on two renowned online shopping sites. All of the participants followed food influencers on social media platforms. Data collection was conducted via an online questionnaire. Ethical practices were observed in accordance with the World Medical Association Declaration of Helsinki, and the protocol was approved by the Ethics Committee of the last author's university. Participants provided informed consent before participating in the study.

### Measures

Socio-demographic characteristics (i.e., age, gender, and subjective social status) were asked. The single-item 10-rung ladder (1 = bottom rung, 10 = top rung) *MacArthur Scale of Subjective Social Status* (*MSSS*) was administered to evaluate respondent’s perception of their place in the social hierarchy [7].

The *Parasocial Relationship with Favorite Food Influencer Scale (PSRFFIS)* was developed by considering conceptual and empirical foundations of parasocial relationships, measures of audience’s parasocial engagement with media figures [3], and previous scales assessing social media users’ parasocial bonds with social media influencers (e.g., Instafamous influencers) [8]. Eight items were created to assess social media users’ feelings of mutual awareness, attention, and adjustment with a food influencer on social media (e.g., “My favorite food influencer makes me feel comfortable, as if I am with friends”). Items are rated on a 5-point Likert scale (1 = *strongly disagree*, 5 = *strongly agree*).

Participants responded to the *Short Form of the Eating Disorder Examination Questionnaire* (*EDE-QS*) to capture eating disorder symptom severity (e.g., “Have you had a strong desire to lose weight?”) [9]. The *EDE-QS* is a unidimensional self-administered 12-item measure. Items are rated on a 4-point Likert scale ranging from 0 (*no day* for items 1–10 and *not at all* for items 11 and 12) to 3 (*6–7 days* for items 1–10 and *very significantly* for items 11 and 12).

The unidimensional 13-item *Modified Yale Food Addiction Scale 2.0* (*mYFAS 2.0*) derived from *Yale Food Addiction Scale 2.0* was used to assess symptoms of addictive eating behavior (e.g., "I ate to the point where I felt physically ill") with eight frequency response options (0 = *never*; 7 = *everyday*) [10].

The *Short Inventory of Grazing* (*SIG*) consists of two items centering on grazing in general and compulsive/loss of control (LOC) grazing [11]. The SIG captures unplanned and repetitious eating of small amounts of food with or without a sense of loss of control. Items are rated on a 6-point scale (0 = *none at all*, 6 = *eight or more times per week*).

Participants' BMI (kg/m^2^) was also asked.

### Procedure

Employing a panel of psychometrics with communication science knowledge (*n* = 6), content validity of the *PSRFFIS* was addressed by computing the Scale-Level Content Validity Index, Averaging Method (S-CVI/Ave*)*. The panel evaluated the relevance of individual items to the underlying construct (I-CVI; 1 = *Not Relevant*, 4 = *Highly Relevant*). The I-CVI was calculated for each item based on the number of experts giving a rating of either 3 or 4 divided by the total number of experts. The S-CVI/Ave score was determined by taking the sum of the I-CVIs divided by the total number of items. An S-CVI/Ave ≥ 0.9 indicates excellent content validity [12]. Factor analysis (exploratory factor analysis––EFA, confirmatory factor analysis––CFA) and item analyses (i.e., corrected item-total correlation, Cronbach’s alpha, and McDonald’s omega value) were computed to evaluate the psychometric properties of the *PSRFFIS*. The overall sample was randomly split in half for EFA (*n* = 202) and CFA (*n* = 203). CFA was performed by applying maximum likelihood estimation to test the factor solution obtained from EFA. Item analyses were conducted on the total sample (*N* = 405). In subsequent analytical steps, the association of sociodemographic characteristics (age, gender, and subjective social status) with PSR with favorite food influencers was explored. Then, the association of PSR with favorite food influencers with dysfunctional eating (i.e., eating disorder, food addiction, and grazing) and BMI were explored by computing Pearson correlation coefficients and multiple regression analysis (*N* = 405). Data analysis was performed using the lavaan package for the software R.

## Results

### Psychometric investigation of *PSRFFIS*

A high S-CVI/Ave of 0.91 was obtained for the eight items, indicating robust content validity of the *PSRFFIS*. Besides, Cronbach’s alpha (*α* = 0.94, 95% CI = [0.93, 0.95]) and McDonald’s omega value (*ω* = 0.94) of *PSRFFIS* were high, and items showed sufficiently high corrected item-total correlation (see Table 1).

**Table 1:** Descriptive statistic, corrected item-total correlation, communality, and factor loadings for *PSRFFIS*

Items	M^*^	SD^*^	Corrected item-total correlation*	${{\varvec{h}}}^{2}$^**^	Factor loading^**^
1.I look forward to watching my favorite food influencer post on their channel(s)	2.13	1.20	0.69	0.43	0.66
2. I would like to meet my favorite food influencer in person	1.68	1.08	0.80	0.72	0.85
3. If there was a story about my favorite food influencer in a newspaper, magazine, website, or social media channel, I would read it	1.94	1.13	0.76	0.59	0.77
4. My favorite food influencer makes me feel comfortable, as if I am with friends	1.60	1.04	0.83	0.71	0.84
5.I think my favorite food influencer is like an old friend	1.57	1.04	0.81	0.77	0.88
6. My favorite food influencer makes me feel as if I am with someone I know well	1.53	0.95	0.80	0.78	0.88
7. I have been seeking out information in the media to learn more about my favorite food influencer	1.67	1.10	0.83	0.75	0.87
8. I am sympathetic toward my favorite food influencer	1.69	1.10	0.83	0.74	0.86

*N = 405, **n = 201.${h}^{2}$=communality, Factor loading = Factor loading from EFA. Item responses ranged from 1 = *strongly disagree* to 5 = *strongly agree*. The *PSRFFIS* was originally developed in Persian, and the English version presented here is translated

After checking item characteristics, an EFA with the Principal Axis Factoring (PAF) method was conducted on a random split-half sample to gauge dimensionality of the *PSRSFIS* with eight items (*n* = 201). The Kaiser–Meyer–Olkin (KMO) value was 0.933 indicating data suitability for structure detection. Moreover, Bartlett’s test was statistically significant (${\chi }^{2}$[28] = 1411.91, *p* < 0.001), demonstrating that EFA with PAF method was applicable. All eight items loaded on one factor through large initial eigenvalue of 5.81 with high communality (${h}^{2}$) and factor loading values, and 68.87% of the total variance explained. CFA with ML method was subsequently conducted with the other random split-half sample (*n* = 203) to confirm the psychometric appropriateness of the one-factor solution, and results indicated an excellent model fit (*χ*^2^** = **20.049, df = 11, *p* < 0.001, CFI = 0.993, TLI = 0.993, RMSEA = 0.064; 90% CI = [0.010, 0.107], and SRMR = 0.018).

### Demographic correlates of PSR with favorite food influencers

Gender-specific variation in PSR with favorite food influencers was investigated via employing independent samples *t *test. Results showed a significant difference in *PSRSFIS* scores between men and women (*t*[342.29] =  − 2.37, *p* < 0.05). Men had higher levels of PSR with their favorite food influencers compared to women.

A multiple regression analysis was carried out to investigate the effect of age, gender, and subjective social status on PSR with favorite food influencers. The composite score, an average of the 8 items of the *PSRSFIS,* was applied as a dependent variable. The minimum age (i.e., 18) was subtracted from each age value to get a meaningful intercept and make interpretation clearer. The multiple regression analysis disclosed that the composite score was significantly related with gender (*B* = − 0.19, *SE* = 0.10, *β* = − 0.10, *p* < 0.05) and subjective social status (*B* = 0.05, *SE* = 0.03, *β* = 0.10, *p* < 0.05), but not with age (*B* = − 0.01, *SE* = 0.01, *β* = − 0.09, *p* > 0.05). The total variance explained by these demographics was 3%. This result means that women had lower *PSRSFIS* composite scores than men. Also, as standard deviation increases in subjective social status, the *PSRSFIS* composite score is expected to increase by 0.10, indicating with the increase in subjective social status, the level of PSR with one’s favorite food influencers increases.

### PSR with favorite food influencers as a predictor of eating disorder, food addiction, grazing, and BMI

In the last phase, the association of PSR with favorite food influencers with eating disorder, food addiction, grazing, and BMI was explored through Pearson correlations and multiple regression analyses. Initially, Pearson correlational analysis revealed weak and moderate, positive correlations of PSR with favorite food influencers with other study-variables, ranging from 0.13 to 0.41 (*r*_*Eating disorder*_ = 0.33, *p* < 0.001; *r*_*Food addiction*_ = 0.41, *p* < 0.001; *r*_*Grazing*_ = 0.22, *p* < 0.001; *r*_*BMI*_ = 0.13, *p* < 0.01). After controlling the effects of gender, age, and subjective social status in multiple regression analysis, PSR with favorite food influencers was significantly associated with eating disorder ($\beta$=0.35, *p* < 0.001), food addiction ($\beta$=0.40, *p* < 0.001), grazing ($\beta$=0.21, *p* < 0.001), and BMI ($\beta$=0.14, *p* < 0.01; see Table 2).

**Table 2:** The predictor role of *PSRFFIS* in food addiction, eating disorder, grazing, and body mass index

Outcome variable	Predictors	*B*	*SE*	$\beta$	${R}^{2}$
Eating disorder	Age	0.00	0.00	0.04	0.12
	Gender	0.07	0.07	0.05	
	Subjective Social Status	− 0.02	0.04	− 0.05	
	PSRFFIS	0.27***	0.04	0.35	
Food addiction	Age	0.01	0.01	0.03	0.18
	Gender	− 0.26	0.14	− 0.09	
	Subjective Social Status	0.04	0.04	0.04	
	PSRFFIS	0.64***	0.07	0.40	
Grazing	Age	− 0.01	0.01	− 0.04	0.06
	Gender	− 0.23	0.16	− 0.07	
	Subjective Social Status	0.01	0.05	0.01	
	PSRFFIS	0.35***	0.08	0.21	
BMI	Age	0.10**	0.03	0.15	0.04
	Gender	− 1.22	0.66	− 0.10	
	Subjective Social Status	0.05	0.19	0.01	
	PSRFFIS	0.95**	0.34	0.14	

**p < 0.01, ***p < 0.001*; N* = 405

## Discussion

There exists limited research on the impact of PSR with food influencers on disordered eating. Moreover, there is a lack of research examining media-related factors contributing to maladaptive eating behaviors in Middle Eastern countries such as Iran, where eating disorders are notably prevalent [6]. This brief report presents preliminary findings on the role of parasocial relationships with food influencers in disordered eating behaviors in an Iranian population.

To quantify PSR with favorite food influencers, a straightforward self-report assessment was constructed and validated. The *PSRFFIS* showed strong psychometric properties. This concise scale can be a valuable tool for future research to assess users’ PSR with favorite food influencers and raise awareness about the psychological correlates of PSR with food media figures. The PSRFFIS can assist researchers to gain a deeper understanding of the interplay between parasocial relationships and eating habits in a social media context.

Men and individuals with higher subjective social status exhibited a greater level of PSR with their favorite food influencers. The results also revealed that stronger PSR with a favorite food influencer predicts more symptoms of eating disorders, food addiction, and grazing. Recent studies [4] have reported that PSR can play a key role in followers' attitudes and behavioral intentions. In specific, influencers promoting food were able to shape followers’ eating habits [2]. In line with these findings, the present results suggested that stronger attachment to a food influencer was associated with stronger cravings for food and more eating problems. These results underscore the importance of considering the role of food influencers in eating problems, particularly in Middle Eastern countries like Iran, where EDs are prevalent, contributing to serious personal and societal problems. The constant exposure to food in such videos, the positive portrayal of disordered eating behaviors (e.g., mukbang watching), and the socio-emotional attachment to the influencer—that is closely associated with wishful identification—can possibly exacerbate maladaptive eating attitudes in some followers.

### Strengths and Limits

This study uses a convenience sample, which limits the generalizability of the findings. Furthermore, the cross-sectional research design employed in the current study prevents the determination of the direction of association between eating disorders and PSR. It is plausible that social media users with pre-existing eating problems are more susceptible to developing a stronger emotional attachment to their favorite food influencer. In this investigation, no other sociodemographic factors (e.g., education level) or the intensity of social media use were examined. Future studies that explore a broader range of socioeconomic and demographic factors may offer a more detailed picture of the role of PSR in eating behaviors. In addition, capturing social media use routines in future PSR studies involving food influencers could provide more nuanced findings by distinguishing between attachment to social media and attachment specifically to an influencer. Moreover, the investigation was conducted in an adult social media user sample. Previous research has found a higher prevalence of dysfunctional eating behaviors among adolescents compared to adults [13]. In addition, age-specific factors, such as hormonal influences, can also impact dysfunctional eating patterns [14]. Therefore, future studies focusing on adolescent users could provide more insights into the relationship between PSR with a favorite food influencer and eating behaviors. However, dysfunctional eating patterns may not be clearly diagnosable in some cases due to the lack of clearly identified symptoms and behavioral patterns, which further limits the investigation of correlates, especially among adolescents. Despite these limitations, this study introduces a robust scale measuring PSR with food influencers and provides preliminary evidence regarding the association of PSR with food influencers with maladaptive eating behaviors.

## Conclusion

The present findings point out that food influencers have responsibility in the portrayal of food and eating attitudes in their videos. The exposure to food and disordered eating behaviors may affect vulnerable followers’ eating attitudes. Therefore, food influencers can play a crucial role in either facilitating adaptive or contributing to maladaptive eating routines and habits among their followers.

## Data Availability

The datasets used and/or analyzed during the current study are available from the first author on reasonable request.
